# HEALTH BEYOND DISEASE IN OLD AGE: RELATIONSHIP OF INTRINSIC CAPACITY WITH HEALTH CARE UTILIZATION

**DOI:** 10.1093/geroni/igad104.1451

**Published:** 2023-12-21

**Authors:** Yafei Si

**Affiliations:** UNSW Sydney, Sydney, New South Wales, Australia

## Abstract

In contrast to the traditional disease-centered approach, intrinsic capacity (IC) is a fine-grained and comprehensive measure of overall health. It plays an essential role in the healthy aging model proposed by the World Health Organization. However, how IC contributes to healthcare utilization and medical expenditures beyond the presence of chronic conditions in old age is not well understood. Data were from the China Health and Retirement Longitudinal Study. We used a validated, multidimensional tool (locomotor, cognitive, psychological capacity, sensory capacity, and vitality) for assessing IC at baseline (Wave 2011). Outcomes include outpatient and inpatient use, and their expenditure measured in Wave 2013. We used logistic and linear regression models to identify the association of IC with healthcare utilization, adjusting for demographics, lifestyles, and multi-morbidity (the presence 2+ out of 14 chronic conditions). We included 14,887 participants, and equally categorized them into three groups (low, intermediate, and high IC). Compared with those with a low IC, older adults with an intermediate level or high level of IC had a significant reduction of outpatient use (OR=0.80, 95% CI=0.72-0.90; OR=0.61, 95% CI=0.53-0.69) and inpatient use (OR=0.69, 95% CI=0.58-0.81; OR=0.58, 95% CI=0.49-0.70), after adjusting for demographics, lifestyles, and multi-morbidity. We did not find a significant association between IC and healthcare expenditure. Assessment of IC for older adults provides valuable information for healthcare use beyond chronic conditions. Assessment of IC in routine clinics may help screen for older adults with high healthcare utilization, which could optimize the allocation of healthcare resources and improve patient-centered care.

